# Regulation of Skeletal Muscle Satellite Cell Differentiation by Omega-3 Polyunsaturated Fatty Acids: A Critical Review

**DOI:** 10.3389/fphys.2021.682091

**Published:** 2021-06-03

**Authors:** Peter O. Isesele, Vera C. Mazurak

**Affiliations:** Division of Human Nutrition, Faculty of Agricultural, Life and Environmental Sciences, University of Alberta, Edmonton, AB, Canada

**Keywords:** inflammation, myogenesis, omega-3, satellite cell, skeletal muscle

## Abstract

Skeletal muscle is composed of multinuclear cells called myofibres, which are formed by the fusion of myoblasts during development. The size of the muscle fiber and mass of skeletal muscle are altered in response to several pathological and physiological conditions. Skeletal muscle regeneration is primarily mediated by muscle stem cells called satellite cells (SCs). In response to injury, these SCs replenish myogenic progenitor cells to form new myofibers to repair damaged muscle. During myogenesis, activated SCs proliferate and differentiate to myoblast and then fuse with one another to form muscle fibers. A reduced number of SCs and an inability to undergo myogenesis may contribute to skeletal muscle disorders such as atrophy, cachexia, and sarcopenia. Myogenic regulatory factors (MRF) are transcription factors that regulate myogenesis and determines whether SCs will be in the quiescent, activated, committed, or differentiated state. Mitochondria oxidative phosphorylation and oxidative stress play a role in the determination of the fate of SCs. The potential activation and function of SCs are also affected by inflammation during skeletal muscle regeneration. Omega-3 polyunsaturated fatty acids (PUFAs) show promise to reduce inflammation, maintain muscle mass during aging, and increase the functional capacity of the muscle. The aim of this critical review is to highlight the role of omega-3 PUFAs on the myogenic differentiation of SCs and pathways affected during the differentiation process, including mitochondrial function and inflammation from the current body of literature.

## Introduction

Skeletal muscle occupies about 40% of total body weight and is a highly dynamic tissue (Frontera and Ochala, 2015). Skeletal muscle accounts for 30–50% of whole-body protein turnover and stores and utilizes substrates, including amino acids and carbohydrates (Frontera and Ochala, 2015). Skeletal muscle is composed of multinuclear cells called myofibers (Fukada, 2018), which are formed by the fusion of myoblasts during development (Yin et al., 2013). The size of the muscle fiber and skeletal muscle mass are altered in different pathological and physiological conditions (Schiaffino et al., 2013). When the muscle is injured, it responds by activating a complex response leading to repair and regeneration of the injured tissue (Tidball, 2011; Barthélémy et al., 2018; Morgan and Partridge, 2020). Skeletal muscle regeneration is primarily mediated by satellite cells (SCs; Lepper et al., 2011; Yamamoto et al., 2018), which replenish myogenic progenitor cells and differentiate into new myofiber for muscle repair in response to injury (Relaix and Marcelle, 2009; McCarthy et al., 2011; Fukada, 2018). SCs are positioned to receive signals from the surrounding environment (Dumont et al., 2015; Fukada, 2018). SCs represent between 2 and 10% of total myonuclei per muscle fiber, and they have self-renewal ability and capacity to give rise to functional progeny. A reduction of SC number and/or an inability to undergo myogenesis may contribute to skeletal muscle disorders such as atrophy and sarcopenia (McKenna and Fry, 2017).

**Figure 1:**
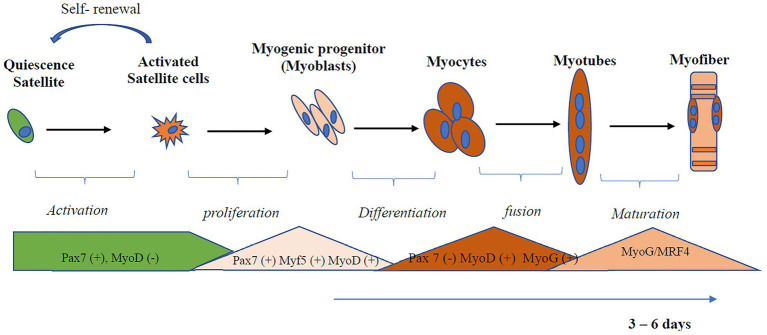
Activation and differentiation of skeletal muscle satellite cell (SC). SCs in an undamaged adult muscle are maintained in a quiescent and undifferentiated state. Quiescent SCs are characterized by the expression of Pax7. Myoblast determination protein 1 (MyoD), a master gene for myogenesis, is expressed at an early stage of myogenic differentiation and induces the expression of other myogenesis-related genes, such as myogenin (MyoG) and MRF4. Myoblast undergoes differentiation and fusion under appropriate conditions from myotubes. *Myf5*, Myogenic factor 5; MyoD, myoblast determination protein 1; MyoG, myogenin; MRF, myogenic regulatory transcription factor; Pax, Paired box protein.

Like stem cells found in other tissues, SCs in an undamaged adult muscle are maintained in a quiescent and undifferentiated state (Montarras et al., 2013). Myogenic regulatory factors (MRF) are transcription factors that regulate myogenesis and determine whether SCs will be in the quiescent, activated, committed, or differentiated state (Olguin and Olwin, 2004; Hernández-Hernández et al., 2017; Zammit, 2017). Transcription factors from the Paired box gene family, *Pax3* and *Pax7*, are critical for satellite cell biogenesis, survival, and potentially self-renewal (Collins et al., 2009). Myoblast determination protein 1 (*MyoD*) is the master gene for myogenesis, is expressed at an early stage of myogenic differentiation, and induces the expression of other myogenesis-related genes such as myogenin (*MyoG*) and *MRF4* (Kassar-Duchossoy et al., 2004; Przewoźniak et al., 2013). MyoD interacts with several metabolic genes involved in mitochondria biogenesis, fatty acid oxidation, and electron transport chain function. The process of myogenic differentiation is illustrated in Figure 1. Quiescent SCs have a minimal number of mitochondria, assessed by reduced levels of mitochondria DNA (mtDNA) tightly packed around the nucleus (Latil et al., 2012). Activated SCs, compared to quiescent SC, rely more on the mitochondria to produce ATP through β-oxidation and oxidative phosphorylation (Ryall et al., 2015). In addition to the role of the mitochondria, inflammation also plays a role in skeletal muscle regeneration (Perandini et al., 2018; Yang and Hu, 2018). The transcription factor, nuclear factor-kappa B (NF-κB), activated by tumor necrosis factor (TNF-α), induces signals that attracts muscle stem cells to the damaged site and promotes proliferation of SCs (Peterson et al., 2011). Myogenesis is also regulated by interleukin (IL)-6 dependent activation of signal transducer and activator of transcription (STAT3; Serrano et al., 2008).

The proliferation and differentiation of SCs can be modulated by nutrients such as dietary carbohydrate, vitamin D, and Vitamin E (Chapalamadugu et al., 2009; Alami-Durante et al., 2011; Khor et al., 2016; Braga et al., 2017); however, their effect varies depending on the origin of muscle type (Powell et al., 2014). Omega-3 polyunsaturated fatty acids (PUFAs) have been shown to increase the functional capacity of the muscle and enable maintenance of muscle mass in aging and chronic disease (reviewed by McGlory et al., 2019; Lam et al., 2020). Omega-3 PUFAs have the potential to regulate the complex process of skeletal muscle regeneration and differentiation due to their ability to regulate gene expression (Price et al., 2000), mitochondria biogenesis (Katyare and Mali, 2016), and inflammation (Calder, 2010). Dietary omega-3 PUFAs, eicosapentaenoic acid (EPA; 20:5n-3), and docosahexaenoic acid (DHA; 22:6n-3) have been shown to be involved in the myogenic program of SCs (reviewed by Bhullar et al., 2016) and skeletal muscle regeneration (reviewed by Tachtsis et al., 2018). However, little is known about how omega-3 PUFA regulates mitochondrial function and inflammation during myogenic differentiation. A recent review (Bhattacharya and Scime, 2020) highlights how mitochondrial function and dynamics affect oxidative phosphorylation and the production of reactive oxygen species to directly impact SC differentiation. The aim of the present review is to consolidate studies that have applied omega-3 PUFA under experimental conditions to study myogenic differentiation that have been published since the review by Bhullar et al. (2016). Since that time, the emerging literature has focused on transcriptional factors regulating myogenesis; this review will evaluate studies exploring the effect of omega-3 PUFAs on the transcriptional regulation of myogenesis.

## Search Method

**Figure 2:**
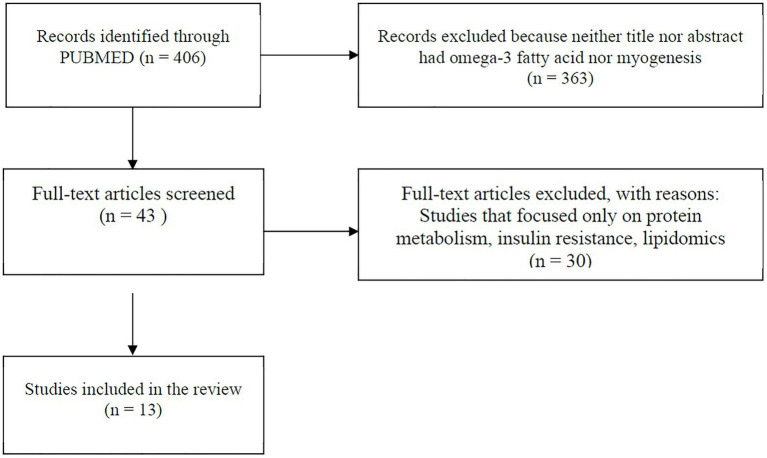
Flowchart showing search method. Papers included were those published between January 2015 and January 2021.

A comprehensive literature search was carried out using PUBMED MEDLINE database. The search included studies published between January 2015 and January 2021. The following keywords were used for literature search: omega-3 PUFAs, eicosapentaenoic acid (EPA), docosahexaenoic acid (DHA), satellite cell, skeletal muscle stem cell, stem cell, AND skeletal muscle, and myogenesis. The search was limited to articles written in English and with full text available. Research articles were screened by title and abstract, and a hand search of reference lists of review articles was performed for relevant studies before they were excluded. Studies were included and considered relevant if they were original articles with omega-3 PUFA intervention and a measure of myogenic differentiation-associated parameters (myogenesis, mitochondria function, or inflammation). Studies were excluded if skeletal muscle cells were not used and if they focused only on protein metabolism, insulin resistance, lipidomic profile, or other pathways unrelated to myogenesis. The information extracted from each study includes model type used, differentiation media, duration of differentiation, treatment (dose), treatment duration, myogenic parameters (myogenesis, mitochondria function, and inflammation), and findings (Table 1). The search strategy is shown in Figure 2.

**Table 1:** Summary of studies that investigated the effects of EPA and DHA on myogenic differentiation.

Reference	Differentiation period	Treatment (dose)	Treatment (duration)	Myogenesis	Mitochondria function	Inflammation	Findings
C2C12 cells							
Chen et al., 2016	6 days	750 μM PA or 50 μM EPA and DHA, AA	24 h	NM	NM	IL-6 and TNF-α, NF-kB, AP-1, mRNA	↓ PA-induced proinflammatory cytokine expression and NF-κB activation
Jeromson et al., 2018	72 h	50-μM EPA and 50-μM DHA	48 h	NM	OCR, function [UCQR2 (complex V and complex III)]	NM	↔ OCR, UCQR2
Hsueh et al., 2018	3 days	50 μM EPA and 50-μM DHA	Fatty acids added to the differentiation media	Myotube number, diameter, Gene expressions (MRF4, MyoD, and MyoG, Pax7)	OCR, Gene expression (ERRα, Tfam, and Pgc-1α, Mitofusin2), mtDNA: nDNA	NM	↓ Myotubes diameter, *MRF4*, *MyoD*, and *MyoG*, and *Pax7* was tended to be suppressed ↓ mtDNA: nDNA, Tfam, Pgc-1α, OCR
Lacham-Kaplan et al., 2020	48–120 h	50 μM EPA	Fatty acids added to the differentiation media	Myotube fusion index, gene expression (MyoD1, MyoG, Myh1, Tmem8c)	NM	NM	↓ Lowest fusion index *↓ MyoD1, MyoG, Myh1, Tmem8c*
Kim et al., 2019	7 days	50-μM EPA	18 h	NM	OCR, basal respiration, proton leak	NM	↓ OCR, basal respiration, proton leak
Lee et al., 2016	5 days	50 μM EPA or DHA	24 h	NM	mRNA levels of Pgc-1α, NRF1, d mtDNA copy number	NM	↑ mtDNA copy number, *Pgc-1α, NRF1*, and *Tfam*
Pinel et al., 2016	5 days	500 μM PAL + 50 μM EPA	16 h	NM	Gene expression (Cpt1α, pgc1α)	NM	↑ *Cpt1α*, ↔ *Pgc1α*
Saini et al., 2017	36 and 72 h	50 μM EPA	Fatty acids added to the differentiation media	Myotube formation, gene expression (MyoD, MyoG)	NM	NM	↑ *MyoD*, ↔ *MyoG*
Shin et al., 2017	4 days	25 μM or 50 μM DHA	24 h	Myotube size and protein expression (MyoD)	NM	NM	Recovered to have the basal levels of *MyoD*
Young et al., 2021	4 days	100 μM EPA, and DHA	48 h	NM	OCR	NM	↓ OCR
Human Myoblast							
Løvsletten et al., 2018	6–7 days	100 μM EPA	24 h	NM	OCR, proton leak	NM	↑ OCR, proton leak
Bovine (MDSCs)							
Xu et al., 2018	48 h of treatment	50 mM DHA	Fatty acids added to the differentiation media	Myotube fusion rate, protein expression (MyoG, MyH3)	NM	NM	↑ Myotube fusion rate, ↑ myotube length, MyoG, and MYH3

All studies that used C2C12 cells were cultured in DMEM + 2% horse serum, except Shin et al. (2017), Løvsletten et al. (2018), and Kim et al. (2019) that used DMEM + 2% FBS, αMEM +2% horse serum, and DMEM + 5% horse serum, respectively. *DHA*, docosahexaenoic acid; DMEM, Dulbecco’s Modified Eagle Medium; EPA, eicosapentaenoic acid; FBS, *fetal bovine serum;* IL, Interleukin; mtDNA, mitochondrial DNA; *Myf5,* Myogenic factor 5; MyoD, myoblast determination protein 1; MyoG, myogenin; MDSCs, muscle-derived satellite cells; MRF, myogenic regulatory transcription factor; NM, not measured; nDNA, nuclear DNA; OCR, oxygen consumption rate; PAL, palmitate; Pgc-1α, peroxisome proliferator-activated receptor-gamma co-activator 1-alpha; Pax, Paired box protein; TNF-α, tumor necrosis factor; TFAM, mitochondrial transcription factor A; Tmem, transmembrane protein; ↓ downregulation; ↑ upregulation; ↔ no change. All studies used DMEM and 2% horse serum as myogenic differentiation media.

## Experimental Models Used to Explore the Role of Omega-3 PUFA on Myogenic Differentiation

The majority of studies (11 out of 13) used the C2C12 cell model. The C2C12 model is the most widely used model to understand molecular mechanisms of muscle differentiation and myogenesis (Cornelison, 2008). Initially, proliferating myoblasts differentiate into elongated myocytes that migrate, adhere, and fuse to one another to form small nascent myotubes (early differentiation) that contain few myonuclei. Nascent myotubes further fuse with additional myocytes or with other myotubes (terminal differentiation/maturation) to generate mature myotubes that contain many myonuclei, which then align with one another into myofibers (Abmayr and Pavlath, 2012). This mimics the process of human muscle satellite cell activation and differentiation in response to muscle injury (Almeida et al., 2016). Loss of Pax7 expression and an increase in MyoG expression characterizes a committed myoblast (Seale et al., 2000; Olguin and Olwin, 2004). The biological and molecular processes of SC activation, proliferation, differentiation, and cell fusion underline the process of skeletal muscle regeneration (Zammit, 2017). Myogenic differentiation media are frequently used to differentiate myoblasts. EPA and/or DHA is added to the media at any point along the differentiation process. Studies that differentiate myoblasts for 3–6 days and subsequently treat myotubes with EPA and/or DHA for 24 or 48 h aim to understand terminal maturation and fusion of the myotube. Most study designs include treatment of skeletal muscle cells with both EPA and DHA and few studies treated with EPA or DHA alone (Saini et al., 2017; Løvsletten et al., 2018; Kim et al., 2019; Lacham-Kaplan et al., 2020).

Under normal conditions, proliferation and differentiation of SCs occur in response to injury and inflammation. However, in most of the studies reviewed, differentiation was induced by a differentiation agent in the absence of injury-related conditions. The control condition applied in most of these studies is cells receiving only differentiation media, which has a much different nutrient composition than human serum. When specific fatty acids are added to the media, this provides additional substrates and nutrients that are not accounted for when the control group has no added fatty acids, posing a limitation in the interpretation of the findings. The mere presence of fatty acids *per se* in the treatment group could evoke responses and add an additional confounding factor to the experimental design. No studies used a control group with added fatty acids of any kind. Aligning the concentrations of serum components to physiological conditions would enhance the translatability of the studies to humans. The concentrations used in the studies ranged from 25 to 100 μM of single or combined fatty acids, which aligns with the concentration of total plasma EPA and DHA in healthy young Canadians; 40 and 88.88 μM of EPA and DHA, respectively (Abdelmagid et al., 2015). Most studies did not report the level of incorporation of fatty acids during differentiation (Table 1).

## Effects of Omega-3 PUFA on Myogenic Differentiation

### Differentiation of C2C12 Myoblasts and Treatment With EPA and DHA

Quiescent SCs are characterized by the expression of Pax7 (Seale et al., 2000). One study reported suppression of *Pax7* expression by EPA+DHA treatment compared to control (Hsueh et al., 2018), suggesting that EPA+DHA induces a transition from quiescent to activated SCs. Hsueh et al. (2018) investigated the effect of EPA + DHA on the relative expression of genes regulating terminal differentiation of myoblast into mature multinucleated myotubes. Downregulation of expression of *MRF4*, *MyoG*, and *MyoD* mRNA was associated with fewer myotubes with a smaller diameter in cells cultured with EPA + DHA compared to control (Hsueh et al., 2018). Myoblast proliferation and gene/protein expression of *MyoG* were reduced at both 48 and 72 h with EPA and DHA treatment (Zhang et al., 2019). In a similar study, using EPA only at the same concentration (50 μM) for 48 h resulted in significantly fewer myotubes, lower fusion index, and reduced expression of *MyoD*, *MyoG*, and *Tmem8c* (myoblast fusion related gene) compared to the control vehicle (Lacham-Kaplan et al., 2020). However, increased expression of *Myf6* (*Mrf4*) and *Myf5* was observed. *Tmem8c* is essential for membrane fusion and myotube extension (Gamage et al., 2017). This suggests that the treated cells were committed to the myogenic lineage; however, the cells had limited ability to fuse to form tubes as EPA suppressed expression of *Tmem8c*. The addition of EPA to the media during the differentiation process (72 h) resulted in higher gene expression of *MyoD*, but no change in *MyoG* compared to the control (Saini et al., 2017).

### Differentiation of Bovine-Derived Myoblast and Concurrent Treatment With DHA

There is only one study that investigated bovine muscle cell differentiation under the influence of DHA alone (Xu et al., 2018). Muscle-derived SCs were isolated from the hind muscle tissues of newborn Chinese Simmental calves, and the differentiating or proliferating myotube was incubated for 2 days with 50 mM DHA (Xu et al., 2018). Longer multinucleated myotubes were formed, and there was a higher myotube fusion rate with DHA treatment compared to control. Similarly, the treatment of the cells with DHA in differentiation media for 48 h increased the expression levels of *MyoG* and *Myh3* compared to the control. However, DHA treatment did not have an effect on the proliferation of the bovine skeletal muscle-derived stem cells (Xu et al., 2018). Therefore, the limited evidence suggests that DHA plays in role in regulating differentiation rather than the proliferation of SCs.

### Treatment of Early Differentiated Myotubes With EPA and DHA

Shin et al. (2017) differentiated C2C12 myoblast for 4 days and then induced muscle atrophy by treatment with dexamethasone as the injury-inducing agent (Shin et al., 2017). After the addition of 25 μM DHA, the atrophied myotubes recovered to having the basal levels of *MyoD* and nucleus number, thereby rescuing the myogenic process. This suggests that the anti-atrophic effect of DHA could be due to the restoration of myogenesis by increasing *MyoD* expression.

**Figure 3:**
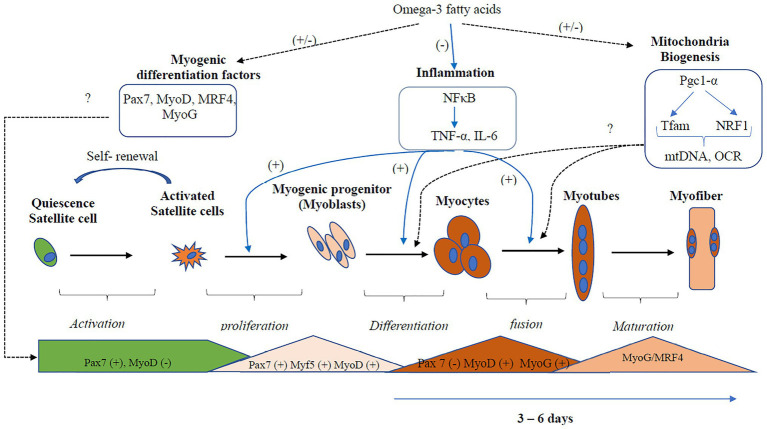
Regulation of inflammation and mitochondria biogenesis by Omega-3 polyunsaturated fatty acids (PUFA) during myogenic differentiation. Omega-3 PUFA inhibits the activation of the inflammatory pathways, thus promoting myogenic differentiation. However, the effects of Omega-3 PUFA on the regulation of the transcription factors that regulate myogenesis and mitochondria biogenesis are inconsistent. Three out of the six studies reported decreased expression, while three studies reported increased expression of myogenic differentiation factors. Two studies reported a decrease in mitochondria biogenesis, while three studies reported decreased expression. Overall, more studies are needed to confirm the actual effect of omega-3 PUFA on mitochondria biogenesis and the transcription factors that regulate myogenesis. Dotted black lines represent pathways that show an inconsistent result, the blue lines represent pathways that show a consistent result. mtDNA, mitochondria DNA; *Myf5*, Myogenic factor 5; MyoD, myoblast determination protein 1; MyoG, myogenin; MRF, myogenic regulatory transcription factor; NF-κB, *nuclear factor kappa B*; OCR, oxygen consumption rate; Pgc-1α, peroxisome proliferator-activated receptor gamma co-activator 1-alpha; Pax, Paired box protein; TNF-α, tumor necrosis factor; Tfam, mitochondrial transcription factor A.

Overall, the effects of EPA and DHA on the formation of myotube and transcriptional regulation of myogenic differentiation are inconsistent (Figure 3). Three out of the six studies reported a decrease in myotube formation and expression of genes involved in myogenic differentiation (*Myf5*, *MyoD*, *MyoG*, and *MRF4*; Hsueh et al., 2018; Zhang et al., 2019; Lacham-Kaplan et al., 2020). On the contrary, three studies (Saini et al., 2017; Shin et al., 2017; Xu et al., 2018) reported an increase in myotube formation and expression of genes involved in myogenic differentiation with EPA or DHA treatment. The reason for the discrepancies in results is not clearly understood. However, Shin et al. (2017) and Xu et al. (2018) reported increased myogenic differentiation by applying a concentration of 25 μM and 50 mM DHA, respectively. In contrast, 50 μM of EPA is generally used in the studies that reported decreased myogenic differentiation with EPA and DHA treatment. One other source of variation in the results obtained by these studies could be the duration of treatment of the C2C12 cells with EPA or DHA. In some studies, the EPA and DHA were added to the differentiation media and present through the period of differentiation days (Saini et al., 2017; Hsueh et al., 2018; Xu et al., 2018; Lacham-Kaplan et al., 2020). In other studies, the cells were differentiated for 3–7 days and then treated with EPA or DHA for 16, 24, or 48 h before investigating myotube formation and expression of genes involved in myogenesis. The timing of exposure to fatty acids is an indicator of which processes are being investigated.

### Endogenous Omega-3 PUFA and Myogenesis in Cardiotoxin-Induced Muscle Injury

Cardiotoxin is an agent used to induce injury in experimental models. Wang et al. (2021) recently examined the role of endogenous omega-3 PUFA on skeletal muscle repair and regeneration following cardiotoxin-induced injury (Wang et al., 2021). In this injury model, *fat-1 mice*, that endogenously produce omega-3 PUFA, had enhanced muscle repair and regeneration. The expression of Pax-7^+^ and MyoD, markers of myogenic differentiation were increased. This indicates a higher rate of satellite cell activation.

## Effects of Omega-3 PUFA on Mitochondrial Function in Differentiating Skeletal Muscle Cell

Mitochondria biogenesis is regulated by the transcription factor peroxisome proliferator-activated receptor gamma co-activator (Pgc-1α), which has the ability to co-activate and augment the expression of several other transcription factors (Fernandez-Marcos and Auwerx, 2011). The expression of nuclear respiratory factors 1 (Nrf1) and mitochondrial transcription factor A (Tfam) is increased by the activation of Pgc-1α (Brown et al., 2010). Nrf1 subsequently upregulates Tfam to stimulate mitochondrial DNA (mtDNA) transcription and replication (Gleyzer et al., 2005). The function of the mitochondria can be assessed by measuring the oxygen consumption rate (OCR; Brand and Nicholls, 2011). Following injury, skeletal muscle-derived SCs increase their OCR and rates of glycolysis (Pala et al., 2018). Five out of eleven studies used C2C12 cells, and one study used human myoblast to measure mitochondrial biogenesis.

### Effects of Omega-3 PUFA on Mitochondria Function in C2C12 Myoblast

Compared to cells cultured in serum alone, mRNA expression of *Tfam* and *Pgc1α* was reduced in myoblasts cultured with EPA and DHA for 3 days (Hsueh et al., 2018). In addition, the ratio of mtDNA to nDNA and the OCR rate was lowered (Hsueh et al., 2018), suggesting fewer mitochondria and reduced mitochondrial biogenesis. Similarly, treatment with 50 μM of EPA for 18 h decreased the OCR, basal respiration, proton leak, and ATP production (Kim et al., 2019). This reveals a negative correlation between high levels of EPA and DHA in the media and the expression of genes that regulate mitochondria biogenesis, suggesting inhibition of mitochondria function and biogenesis in differentiating myoblasts. On the contrary, an increase in the mRNA of *Pgc1-a*, *Nrf1*, and *Tfam* was observed when myotubes were treated with 50 μM EPA+DHA for 24 h, increased mtDNA/nDNA and mtDNA copy numbers were reported (Lee et al., 2016). These results suggest improvement of mitochondria biogenesis and function in the myogenic process with EPA+DHA treatment. While Young et al. (2021) reported increased mitochondrial respiration, another study reported no alteration of mitochondria respiration (determined by OCR) and function [UCQR2 (complex V and complex III)] by treatment of C2C12 myotubes with 50 μM EPA and DHA for 24 h (Jeromson et al., 2018). The difference between these studies could potentially be attributed to the concentration and duration of the treatments, one of the studies treated with 50 μM of the omega-3 PUFA for 24 h (Jeromson et al., 2018), and the other treated with 100 μM for 48 h (Young et al., 2021).

### Effects of Omega-3 PUFA on Mitochondria Function in Human SCs

The role of omega-3 PUFAs on mitochondrial biogenesis pathways in humans is not well-understood. Only one study published in the past 4 years isolated SCs from healthy individuals (Løvsletten et al., 2018). SCs were isolated from muscles of nine healthy donors and induced to differentiate to form multinucleated myotubes. Cells were provided 100 μM EPA for 24 h increased the OCR, basal respiration, proton leak, and maximal respiration compared to palmitic acid. This suggests that the EPA improves mitochondrial function during the myogenic process. In support of this, Yoshino et al. (2016) explored the effect of omega-3 PUFAs (EPA and DHA) supplementation for 6 months on muscle transcriptome in older adults. Omega-3 PUFA supplementation to humans has been shown to upregulate the pathways involved in mitochondria function and extracellular matrix organization, a decrease in ubiquitin-mediated proteolysis, a decrease in pathways involved in the inhibition of mTOR (Yoshino et al., 2016). However, a minimal effect on the expression of individual genes involved in mitochondria was observed (Yoshino et al., 2016), which does not align with *in vitro* models, which show a decrease in OCR and basal respiration (Pinel et al., 2016; Hsueh et al., 2018; Kim et al., 2019). The difference could be attributed to the difference in the concentration used and duration of differentiation.

Overall, there were inconsistencies in the regulation of mitochondria function and biogenesis by EPA and DHA (Figure 3). The mitochondria function and biogenesis were determined by investigating the ratio of mitochondria DNA to nuclear DNA (mtDNA/nDNA), OCR, and the expression of genes involved in mitochondria biogenesis. One study reported no change (Jeromson et al., 2018), two studies reported an increase (Lee et al., 2016; Løvsletten et al., 2018), and three studies reported a decrease (Pinel et al., 2016; Hsueh et al., 2018; Kim et al., 2019) in mitochondrial function with EPA+DHA treatment. The inconsistencies in the experimental design make the study comparison challenging.

## Effects of Omega-3 Fatty Acids on Inflammation During Myotube Differentiation

Only one study measured the effect of EPA and DHA on inflammation during myogenic differentiation (Chen et al., 2016). The transcription factor NF-κB is crucial for the induction of proinflammatory cytokine expression and plays an important role in the pathogenesis of diseases related to chronic inflammation (Martins et al., 2016; Liu et al., 2017). A high concentration of TNF-α plays a role in inducing proliferation of SCs and inhibition of myogenic differentiation through MyoD protein destabilization (Li, 2003; Langen et al., 2004). Inhibiting the action of TNF-α also inhibits the activity of p53 mitogen-activated protein kinases (MAPK), leading to the downregulation of expression of muscle differentiation markers such as MyoD and the transcription factor MyoG (Chen et al., 2007; Zhan et al., 2007). Palmitic acid-induced proinflammatory cytokine expression is associated with the development of insulin resistance in myotubes (Saghizadeh et al., 1996; Wellen and Hotamisligil, 2005). Co-treatment of C2C12 myotubes with 750 μM palmitic acid and 50 μM EPA and DHA for 16 h resulted in an inhibition of palmitic acid-induced proinflammatory cytokine expression (Chen et al., 2016). The expression of TNF-α and IL-6 mRNA was reduced in the EPA and DHA treatment compared to the control. Similarly, EPA and DHA treatment abolished palmitic acid-induced NF-κB activation by decreasing IκB-α degradation, NF-κB nuclear protein DNA binding activity, and NF-κB transcriptional activity. EPA and DHA downregulates NF-κB activation through several mechanisms, one of which is *via* a G-protein coupled receptor GPR120 (Talukdar et al., 2010). While ligand-stimulation of GPR120 led to an increase in glucose transport and translocation of glucose transporter (GLUT4) to the plasma membrane in adipocytes (Talukdar et al., 2010), and in muscle cells (Kim et al., 2015), their role in inflammation and myogenesis in skeletal muscle cells remains uncharacterised (McGlory et al., 2019). In adipocytes, DHA inhibits NF-κB activation and expression of NF-κB target genes and proteins *via* GPR120 (Talukdar et al., 2010). G-protein-coupled receptor kinase 2 (GRK2) phosphorylates the active form of G-protein-coupled receptor (Ribas et al., 2007) and plays a role in myogenesis (Garcia-Guerra et al., 2014); however, how this receptor is modulated by omega-3 PUFA is not known.

## Limitations and Future Direction

SCs play a crucial role in the repair of damaged skeletal muscle fiber in response to injury. An alternate approach to study similar conditions observed with muscle injury-induced inflammation is to monitor these cells during differentiation, coupled with a stimulus that recapitulates muscle injury (e.g., TNF-α or palmitate-induced inflammation). An appropriate control for these studies would be cells that are exposed to a similar amount of total fatty acids, without EPA and DHA, while the treatment group receives similar fatty acids plus EPA and DHA to maintain a similar molar concentration of total fatty acids in the media. Further, this could be further refined to ensure physiological concentrations. This would enable attribution of the effects specifically to EPA or DHA while enhancing the translatability of the findings. *In vivo*, muscle cells are exposed to a mixture of fatty acids. The only study that isolated SCs from human biopsy treated the skeletal muscle cells with 100 μM of EPA (Løvsletten et al., 2018) which is twice the concentration (50 μM EPA or DHA) that were used in most of the studies that used C2C12 cell. Correlating the concentrations used in the *in vitro* studies to physiological values is challenging; hence, it is difficult to extrapolate these results to humans. In addition, most studies did not report the level of incorporation of fatty acids during the differentiation. Future studies are needed to understand how the EPA and DHA modify myotube formation and myogenic differentiation. It is also essential to investigate the effects of omega-3 PUFA on SCs differentiation in cancer patients, to provide more insights on the regulation of skeletal muscle regeneration by EPA and DHA. Further studies should also explore the differentiation potential of SCs in response to stimuli that rehabilitate muscle injury and how exposure to EPA to DHA will alter the myogenic differentiation of the SCs. Future studies should focus on also examining whether omega-3 PUFA meditation of inflammation during satellite cell differentiation is *via* G-protein-coupled receptor. The physiological relevance of these *in vitro* studies remains unclear.

## Conclusion

In physiological conditions, there is a complex interaction between molecules in the regulation of pathways involved in myogenesis, hence the need for more studies to investigate this process. Most studies have reported beneficial effects of omega-3 PUFA in the skeletal muscle of cancer patients; however, the mechanism is not fully understood. Myogenic differentiation of satellite cells into myotube and further fusion into muscle fiber is one potential mechanism during skeletal muscle regeneration. There is a need for more studies to explore the effects of omega-3 PUFAs on human SCs myogenesis to get more insights on skeletal muscle regeneration during muscle injury and maintenance of skeletal muscle mass.

## Author Contributions

PI drafted the manuscript. VM edited and revised the manuscript. Both the authors contributed to the article and approved the submitted version.

### Conflict of Interest

The authors declare that the research was conducted in the absence of any commercial or financial relationships that could be construed as a potential conflict of interest.
